# Tuberculosis-Associated Anemia: A Narrative Review

**DOI:** 10.7759/cureus.27746

**Published:** 2022-08-07

**Authors:** Thejaswi Dasaradhan, Jancy Koneti, Revanth Kalluru, Sai Gadde, Swathi priya Cherukuri, Rahul Chikatimalla

**Affiliations:** 1 Research, Narayana Medical College, Nellore, IND; 2 Research, Kamineni Institute of Medical Sciences, Narketpally, IND

**Keywords:** antitubercular therapy (att), cytokines, iron therapy, ferritin, hepcidin, anemia of chronic diseases, anemia, tuberculosis

## Abstract

Tuberculosis (TB) is an airborne illness that induces systemic inflammation. It often affects the lungs causing cough, fever, and chest pain. A commonly associated comorbid condition in TB is anemia. This review article has summarized various studies with an aim to gain a better understanding of pathogenesis and the role of cytokines that contribute to the development of anemia in TB. The study has gathered risk factors that enhance the likelihood of TB patients acquiring anemia. It has reviewed therapeutic modalities such as antitubercular therapy and iron therapy in an attempt to find which of them are effective in reducing the severity of anemia. This review article has also emphasized the importance of measuring hepcidin and ferritin and has touched upon the investigations that can be easily implemented.

## Introduction and background

TB is an airborne illness caused by Mycobacterium tuberculosis (MTB), which often affects the lungs and causes severe coughing, fever, and chest pain [1]. In 2019, TB remained as the leading cause of death due to a single infection, and an estimated 10.0 million persons worldwide contracted TB [2]. South-East Asia and African areas continued to have the largest number of people infected with TB, and in the European region, a decrease in TB infection was on track to meet the 2020 targets [3]. The End Tuberculosis Strategy, a World Health Organization (WHO) initiative, set ambitious targets for 2020-2035, including a 20% reduction in TB incidence and a 35% reduction in the absolute number of TB deaths by 2020, a 90% reduction in TB incidence, and a 95% reduction in TB deaths by 2035 [4]. Risk factors for TB include age, gender, immune status, malnutrition, diabetes, poor ventilation, alcohol, smoking, occupational risk, duration of contact, and proximity with the infected individual [5]. Following aerosol transmission to a new host, MTB is thought to be initially phagocytosed in the lung by alveolar macrophages and dendritic cells, after which it undergoes unrestricted intracellular replication along with infected cells traveling to draining lymph nodes in the surrounding area. After reaching the regional lymph node, MTB disseminates through the circulation and infects additional host cells, eventually re-seeding the pulmonary area. In response to cellular immunity, local pro-inflammatory reactions trigger the recruitment of more monocytes and lymphocytes, which aggregate around infected macrophages to contain MTB inside an organized cellular structure known as a granuloma [6]. The typical clinical symptoms of pulmonary TB (PTB) are chronic cough with sputum production, loss of appetite, weight loss, fever, night sweats, and hemoptysis [7]. Primary PTB is characterized by local granulomatous inflammation in the periphery of the lung called Ghon focus. Up to 25% of TB cases present with extrapulmonary involvement, and common sites involved are lymph nodes, pleura, and the osteoarticular system [8]. Miliary TB is a disseminated type of TB that involves many organs, while latent TB infection is clinically inapparent illness [9]. Some of the investigations employed for the detection of active TB are polymerase chain reaction (PCR), cartridge-based nucleic acid amplification test (CBNAAT), culture, and direct microscopic examination of the pathogen. Specimens required for the diagnosis are sputum and bronchoalveolar lavage (BAL). Imaging techniques commonly used are chest X-ray and computed tomography (CT). The treatment for PTB consists of two-month intensive phase with isoniazid (INH), rifampicin (RMP), ethambutol (EMB), and pyrazinamide (PZA), which is followed by a four-month continuation phase with INH , RMP, EMB [10]. Extrapulmonary and disseminated TB may require more extensive therapy depending on the site of involvement. Lymph node TB requires six months’ therapy, articular or osseous TB requires nine months of therapy, while TB of the central nervous system (CNS) involves therapy for 12 months [11]. Drugs such as amoxicillin, cycloserine, carbapenem, p-aminosalicylic acid, fluoroquinolones, macrolides, clofazimine, and PZA are used for the treatment of multidrug-resistant TB [12].

Anemia is defined as hemoglobin (Hb) levels below 12.5 g/dL for women and 13.5 g/dL for men [13]. Anemia can result in fatigue, as well as reduced cognitive ability and productivity, which lower quality of life [14]. Many patients with active PTB have low Hb, and anemia is the most common comorbidity present in TB. According to some study analysis, anemia is evident in 61.53% of TB patients. The overall prevalence of mild, moderate, and severe anemia is 35.67%, 31.1%, and 11.6%, respectively [15]. Furthermore, the prevalence of anemia of chronic disease (ACD) and iron deficiency anemia (IDA) is 49.82% and 20.17%, respectively. Hb levels are low at the time of diagnosis of TB or during the progression of the infection, which has a direct influence on the treatment outcome and lifestyle of the patient, and also the prevalence of recurrent TB infection is high among individuals with low Hb levels. Anemia can be caused by several reasons, including deficiency of iron and chronic inflammation [16]. This review article aims to show a distinct perspective on the correlation between TB and anemia by discussing the mechanism and pathophysiology of anemia in TB and highlighting the screening guidelines and management of the above-mentioned disease concerning TB.

## Review

Mechanism

Anemia in TB is most often due to nutritional deficiency, malabsorption, and ACD [17]. Low dietary intake is one of the causes resulting in IDA. Loss of appetite, a classic symptom of TB, may be the reason for reduced dietary intake. Severity may deepen with associated co-helminthic infections. Malabsorption problems result in decreased iron absorption and IDA [18]. Intestinal involvement of TB can be primary, resulting from organism ingestion, or secondary from pulmonary source. TB intestine involving the ileocecal region and ulcerative type predominantly present with malabsorption syndrome [19].

TB infection is known to induce systemic inflammation and lung damage. Anemia of chronic illness, also known as anemia of inflammation(AI)/ACD, is a clinical phenomenon defined as the development of anemia in patients with infectious diseases (fungal, bacterial, or viral) such as TB, inflammatory diseases, autoimmune disorders, and neoplastic diseases [20]. AI is caused by inflammation-related pathogenesis such as short erythrocyte life span, poor erythrocyte iron incorporation, and decreased sensitivity to or supply of erythropoietin [21].

Microorganism invasion activates T lymphocytes (CD3+) and monocytes, which initiate immunological effector mechanisms, generating cytokines such as interferon-γ (IFN-𝛶) from activated monocytes and tumor necrosis factor-α (TNF-α), interleukin (IL) such as IL-1, IL-6, and IL-10 from monocytes or macrophages. Lipopolysaccharides (LPS) are a prominent component of gram-negative bacteria's outer membrane and are endotoxins that act as a potent stimulator for innate or natural immunity [22]. At various phases of its intracellular life, MTB controls inflammation, recognition, phagocytosis, replication in the phagosome, and cytosol escape, which result in the regulated release of cytokines such as IL-1, TNF-α, and IL-10, lipid mediators, and IFN-γ [23]. One of the characteristics of ACD is the development of iron homeostasis problems, with increased iron absorption and retention among reticuloendothelial system cells. Therefore, iron is diverted from circulation and stored in the reticuloendothelial system, limiting the availability of iron for erythroid progenitor cells and resulting in iron-restricted erythropoiesis [24]. IFN-γ, LPS, and TNF-α enhance divalent metal transporter 1 (DMT1) expression, resulting in increased iron absorption by activated macrophages. Downregulating ferroportin expression in macrophages prevents iron release from these cells. Ferroportin is an iron transmembrane exporter, which is responsible for the transport of ingested ferrous iron from duodenal enterocytes and macrophages to the circulation [25,26]. LPS and IL-6 induce hepcidin production [27]. Hepcidin levels are increased in systemic inflammation and/or infection, which results in iron dysregulation, hypoferremia, and anemia from inflammatory illness [28]. In inflammatory situations, hepcidin production is no longer regulated by iron load, that is, if iron levels have dropped, hepcidin synthesis should be downregulated, but it enhances due to IL-6 activation. Hepcidin is a peptide hormone that limits iron entry into the plasma from the three major sources of iron, that is, food absorption in the duodenum, macrophage release of recycled iron, and hepatocyte release of stored iron [29]. Hepcidin prevents iron release from reticuloendothelial macrophages, liver, and duodenum by binding and downregulating ferroportin receptors, leading to reduced dietary absorption and causing its internalization and degradation, which results in sequestration of iron into the reticuloendothelial system [29,30]. Because iron is a key growth factor for MTB, iron retention in the reticuloendothelial system is regarded as one of the host defense strategies [31]. TNF-α, IL-1, and endotoxin, which are substantially generated during inflammatory diseases, contribute to the development of anemia by reducing the lifespan of red blood cells (RBCs) and also by drastically decreasing plasma iron incorporation into freshly produced RBCs.

Acute phase reactants are plasma proteins produced mainly by the liver in response to infection and inflammation. C-reactive protein (CRP) and ferritin are elevated in TB patients with anemia. Elevated ferritin levels do not always indicate iron repletion since this molecule is an acute phase reactant and its expression could be influenced by inflammation [32]. Gil-Santana et al. conducted a study that was published in 2019 where 118 PTB were retrospectively analyzed during the first 60 days of antitubercular therapy (ATT). Prior to therapy, multidimensional statistical analyses were used to study a thorough inflammatory profile of patients stratified by anemia status. Anemia-related diagnostics such as elevated ferritin, CRP, and erythrocyte sedimentation rate (ESR) were identified, which partially reverted at 60 days of ATT. The results suggest that chronic inflammation was most likely to be responsible for the majority of anemia cases [16]. Plasma erythropoietin (EPO) levels are usually low in comparison to blood Hb concentrations in individuals with AI. IL-1 and TNF-α were shown to inhibit EPO mRNA, making anemia more common in TB due to inflammation rather than iron deficiency [33]. Sahiratmadja et al. performed a case-control study that was published in 2007 in Indonesia. They included 378 PTB patients and 436 healthy controls from the same neighborhood and same socioeconomic status in which hematological and iron parameters were measured among them. In TB patients, lower Hb concentrations were associated with lower plasma iron concentrations, lower iron binding capability, and increased plasma ferritin. Iron parameters increased toward control values after effective TB therapy, and Hb levels normalized even without iron supplementation. The majority of active TB patients had anemia, which was most likely due to inflammation rather than iron deficiency, considering that TB therapy alone was sufficient to restore Hb levels [34]. Iron metabolism in inflammatory disease is characterized by tissue iron release restriction, reduced serum iron and total iron-binding capacity (TIBC), and elevated serum ferritin levels. The altered iron metabolism is hypothesized to be a component of the host defense system against invading pathogens, and it is thought to be mediated by inflammatory cytokines [35]. The majority of the drop in transferrin concentration is due to increased protein catabolism [36].

Risk factors

Low body mass index (BMI), HIV infection, helminthic co-infection, low selenium concentrations, old age, high retroviral load, high IL-6 concentrations, and female gender are some of the risk factors associated with anemia in adults with TB. In rat erythrocytes, dietary selenium significantly boosted plasma glutathione peroxidase activity. Accordingly, greater selenium contents in rat erythrocytes were thought to be responsible for improved resistance to oxidative stress. Glutathione peroxidase is an enzyme that aids in the defense against free radicals and oxidative stress [37]. Van Lettow et al. conducted a study that was published in 2005 in the Zomba district for two years. The study population included 500 persons who had PTB, of which 370 were HIV positive and 130 were HIV negative. EPO, IL-6, plasma HIV load, indices of micronutrient status Hb, plasma concentrations of retinol, tocopherol, carotenoids, ferritin, zinc, and selenium were assessed. IL-6 concentration was 21.1 pg/mL in both HIV-positive and negative adults. These studies described that low selenium levels, high HIV load, and IL-6 concentration are associated with an increased risk of anemia in TB patients [38].

Investigation

A thorough history of the disease is required for the initial evaluation. It is necessary to analyze the biochemical profile in TB patients with a goal to identify profiles associated with anemia and their response to antitubercular medication.

Hemoglobin

Hb is a protein that is present in RBCs and anemia tends to occur when a patient's Hb level is low. Anemia is classified into mild, moderate, and severe based on Hb concentration. Mild form corresponds to an Hb level of 10.0/dL to lower limit of normal, moderate form corresponds to Hb level of 8.0 to 10.0 g/dL, and severe form corresponds to Hb level of 6.5 to 7.9 g/dL [39].

Mostly, anemia in TB ranges between the mild and moderate forms. de Mendonça et al. conducted a retrospective cross-sectional study that was published in 2021. In this study, 328 samples were evaluated, of which 70 were rejected and 258 were confirmed. To confirm the relationship between anemia, clinical state, and laboratory data, exploratory and logistic regression analyses were performed. The study revealed that anemia is common in TB, predominantly normocytic normochromic anemia in mild-to-moderate forms, and anemia was severe in disseminated and meningeal TB, suggesting that anemia may be a biomarker of TB severity [40]. Abay et al. conducted a cross-sectional study that was published in 2018 with 50 PTB patients and 50 PTB-HIV co-infected individuals. Around 5 mL of venous blood was taken and split into 3-mL ethylene-diamine tetra acetic acid (EDTA) tubes for complete blood picture and 2-mL citrated tubes for ESR determination. The independent t-test was used to compare the mean values of hematological parameters between PTB and PTB-HIV co-infected patients. Studies have shown that PTB-HIV co-infected individuals had a greater prevalence of anemia, neutropenia, thrombocytopenia as compared to the non-infected ones, as HIV tends to enhance PTB patients’ hematological abnormalities [41]. A frequent hematological abnormality in PTB patients is normocytic normochromic anemia, while microcytic hypochromic anemia is not uncommon. Mukherjee et al. conducted a one-year retrospective study at a tertiary care hospital in Uttarakhand and included all new TB cases above the age of 18 years. Clinical, demographic, and biochemical data were obtained. Of the 252 patients, 181 were anemic and 103 had normocytic normochromic anemia. Therefore, screening for anemia in all TB cases is suggested [42].

Mean Corpuscular Volume, Mean Corpuscular Hemoglobin Concentration, and Mean Corpuscular Hemoglobin

Mean corpuscular volume (MCV) determines the size of RBCs, mean corpuscular hemoglobin (MCH) measures the quantity of Hb in each RBC, and mean corpuscular hemoglobin concentration (MCHC) value represents the quantity of Hb per unit volume [43]. These are called red cell indices, and any abnormalities in these values indicate anemia.

MCV, MCHC, and MCH values are lowered in TB patients, indicating low Hb concentration as the disease progresses chronically. Kahase et al. published a cross-sectional study in 2020. A total of 40 patients with TB and 40 non-TB patients as control were assessed for hematological parameters. Around 5 mL of venous blood and 2-5 mL of sputum were collected and examined by Cell Dyn 1800 hematology analyzer (Abbott Laboratories, Abbott Park, IL, US) and cultured using BACTEC MGIT 960 (Becton Dickinson, Franklin Lakes, NJ, USA). According to the study, Hb, HCT, and MCHC were low in TB patients as compared to controls [44]. Atomsa et al. performed a cross-sectional study that was published in 2020 where Hb, RBC, MCHC, MCV, and WBC were compared between TB patients and healthy controls. CD4 cells, Hb, MCV, MCHC, and mean RBC counts were low in TB patients compared to the controls. Determining hematological parameters is crucial for case management [45]. Decreased MCH and MCV in anemia patients, as well as the prevalence of microcytic hypochromic anemia, lead us to believe that iron nutritional deficiency and decreased availability of iron due to alterations in its metabolism are important causes of iron anemia in chronic infections such as TB [40].

Cytokine Release

Cytokines are tiny proteins generated essentially by every cell in order to control and modulate immune response [46]. Various cytokines are released in response to infection by immune system, and one of the major functions of cytokines is to regulate inflammation. Elevated cytokine levels in patients with anemia are indicative of ACD.

TNF-α, IL-1, and IL-6 are proinflammatory cytokines and lead to immune cell activation and synthesis, as well as the release of more cytokines [47]. IFN-γ and IL-10 are also cytokines raised in inflammatory conditions such as TB [48]. Multiplex immunoassays and enzyme-linked immunosorbent assay (ELISA) are most commonly used to measure plasma cytokines. The population data are shown in Table 1.

**Table 1:** Population data table TB, tuberculosis; TNF-α, tumor necrosis factor-alpha; IFN-γ, interferon-gamma; IL-6, interleukin-6; MIP, macrophage inflammatory protein; MTB, Mycobacterium tuberculosis; ETB, extrapulmonary tuberculosis; ELISA, enzyme-linked immunosorbent assay; VEGF-A, vascular endothelial growth factor-A; COPD, chronic obstructive pulmonary disease

Reference	Number of cases and control	Diagnostic criteria	Conclusion
Xiong et al. [49]	115 TB cases and 107 non-TB cases	Estimation of cytokines	TNF-α, IL-6, IFN-γ, and MIP significantly high in TB cases
Ranaivomanana et al. [50]	THP-1 derived macrophages infected with MTB isolated from TB, ETB	Cytokines measured with the help of ELISA	TNF-α high in TB and VEGF-A high in ETB-infected cases
Tang et al. [51]	152 TB cases with COPD, 150 cases with TB, 157 cases with COPD, and 50 cases of health workers	Cytokines measured using ELISA	IL-6, TNF-α, and IFN-γ were high in TB cases with or without COPD; relatively, the above-mentioned cytokines were much higher in TB patients with COPD

Hepcidin

Hepcidin levels are elevated in inflammatory conditions such as chronic infections, and their levels are significantly increased in patients with ACD [52]. Measuring hepcidin levels in blood or urine aids in the diagnosis of anemia [53]. Serum and urine hepcidin concentrations are considerably lowered in patients with pure IDA [52]. Hepcidin can also be measured using ELISA assay with accuracy [54]. Hella et al. conducted a study that was published in 2018, in which serum samples of 102 TB cases and 93 non-TB controls were assessed and compared for hepcidin, iron, and inflammatory parameters. Hepcidin was higher in TB cases and the five controls who developed TB. Increased hepcidin levels indicate altered iron metabolism [55]. Tashiro et al. conducted a prospective observational study that was published in 2019 in which hepcidin levels of 35 PTB were measured on admission, and the study revealed a significantly higher level of hepcidin in TB patients [56].

Serum Ferritin

Ferritin is a blood protein that has iron within it, and low serum ferritin indicates low iron storage and iron deficiency. Serum ferritin is not only a blood protein but also an acute phase reactant and inflammatory marker that is raised in a variety of inflammatory diseases. Normal acceptable ferritin value ranges between 30 and 300 ng/mL in males and between 10 and 200 ng/mL in females [57]. Increase in ferritin levels in patients with PTB ranges between 500 and 800 ng/dL [58]. Ferritin levels are not much decreased with one month of ATT but substantially after 60 days of ATT [59]. Mostly, anemia resolve by the end of end months of ATT.

Oliveira et al. conducted a descriptive longitudinal study that was published in 2014. The study included 166 TB patients in whom various inflammatory parameters were measured. Ferritin and ESR were high in patients with ACD [58]. Mishra et al. conducted a study with 100 adult TB patients who were compared with 60 healthy individuals in which serum CRP and ferritin were found to be raised [60].

Soluble Transferrin Receptor

The transferrin receptor is a transmembrane cellular protein primarily expressed in cells that require iron. Soluble transferrin receptor (sTfR) level is elevated or unaffected in ACD [61]. Ratnaningsih et al. conducted a cross-sectional study that was published in 2020. The study consisted of three population groups: 68 patients with TB and anemia, 7 patients with IDA, and 15 non-anemic TB patients. Ferritin and sTfR were measured and compared between the study groups. There was no significant difference in sTfR levels in the TB anemic group and non-anemic TB group. Measuring sTfR levels in anemic patients helps in identifying iron deficiency where sTfR is raised [62].

Serum Transferrin and TIBC

Rise in transferrin levels indicates that the body requires more iron. TIBC is an indirect marker of transferrin. Hypoferremia is caused by iron entrapment in the cells of the reticuloendothelial system, and this low serum iron leads to poor transferrin saturation. As a result, transferrin is lowered or normal in ACD [63].

ESR and CRP

ESR and CRP are diagnostic parameters used to assess inflammation. They are raised during both acute and chronic inflammation [60,61]. They are sensitive markers of inflammation but lack specificity.

Treatment

The initial line of treatment for anemia in TB is to subside the underlying inflammatory process. ATT is an approved six-month medication course employed in the treatment of TB. The medications include RMP, INH, PZA, and EMB for two months, and INH, RMP, and EMB for four months [10]. These drugs when given in combination stop the growth of MTB by means of various mechanisms [64]. ATT inhibits the growth of bacteria and decreases the secretion of cytokines, which are synthesized as a response to MTB. ATT for TB is associated with a considerable decrease in AI. Minchella et al. conducted a study that was published in 2015 in which 45 patients with PTB, 47 tuberculin skin test (TST) as positive controls, and 39 TST negative as controls were followed and assessed at the second and sixth months of ATT. Hb, ferritin, hepcidin, transferrin, and sTfR were measured in both cases and controls. Anemia was more common in TB cases than in control groups. Based on the iron biomarkers, anemia was classified into AI, IDA, and multifactorial anemia (IDA + AI). Anemia was more common in 67% of TB patients than in 36% of TST-positive and 21% of TST-negative controls. AI was the most common anemia among TB-diagnosed individuals, and after six months of complete treatment, AI incidence dropped from 36% to 8%. However, a matching reduction in anemia with iron-responsive components (IDA, IDA +A I) was not seen. Therefore, ATT is associated with significant reduction in AI [65]. Lee et al. conducted a study that was published in 2006 over a span of 11 months, where 880 TB patients were included, of which 281 had anemia at the time of diagnosis. Ten patients with iron replacement therapy were excluded from the study. Hb concentrations were measured, and anemia improved in 175 of 271 individuals without iron consumption during or after ATT. Anemia is common in TB patients; therefore, attentive surveillance is adequate because TB-associated anemia is generally moderate and improves with anti-TB medication [66]. Mesquita et al. conducted a study that was published in 2016 in which serum samples of 73 PTB patients were examined for circulatory levels of cytokines such as IL-6, IL-10, IFN-γ, CRP, ESR, and TNF-α at pre-ATT and on day 60 of treatment. The results showed decreased levels of CRP, IL-2, TNF-α, and ESR, and increased levels of IL-10 on initiation of treatment. MTB load in sputum and systemic inflammation are symbiotically associated [67]. Luo et al. conducted a study that was published in 2018 in which immune markers of 232 PTB cases were measured before and after two months of ATT and compared with 50 healthy volunteer controls. Serum levels of IL-1β, IL-6, and TNF-α were measured with the help of ELISA, and concentration of cytokines was found to be decreased after treatment initiation [68]. As in most of the cases, anemia in TB ranges between mild and moderate forms, and ATT alone helps to resolve anemia.

In cases of severe anemia in TB, iron therapy should be considered only after the inflammation subsides. CRP and ESR are the biomarkers of subsided inflammation, which can be measured easily by a simple blood test. Iron therapy when given during the active phase of the disease promotes the proliferation of microorganisms [61]. Since hepcidin blocks iron absorption, iron therapy should be started once the hepcidin levels return to the pre-inflammatory range. Cercamondi et al. conducted a prospective study that was published in 2021 and included 18 TB patients; iron kinetics, iron metabolism indices, and inflammatory changes were studied every two weeks after ATT initiation. Iron tracers were administered at various stages of treatment. The results showed inflammation resolution, fall in hepcidin levels, improvement in the replenished iron stores, and enhanced iron absorption after ATT completion. Therefore, iron therapy is given to individuals with persistent anemia in whom anemia could not resolve with ATT [69].

Limitations

Anemia is a multifactorial disease, and the only risk factor considered in this article is TB as a chronic infection. This article presents overall pathogenesis of anemia in chronic diseases such as TB, as well as important investigations and management strategies for anemia in TB due to chronic inflammation. Additional studies are required to evaluate pathogenesis of IDA and ACD+IDA in TB.

## Conclusions

This review paper discussed the troubling link between TB and anemia. This article aimed to analyze the processes that contribute to the development of anemia and to explore effective therapy to minimize the morbidity of TB-associated anemia. Cytokines and inflammatory markers including TNF-α, IFN-𝛶, hepcidin, and ILs contribute to the development of anemia in patients with chronic diseases. ACD can be diagnosed with different parameters such as Hb, transferrin, hepcidin, TIBC, ESR, CRP, and sTFR. Anemia in TB patients falls between the mild-to-moderate range and tends to resolve after the completion of ATT alone, and treating anemia with iron therapy during the active phase of the disease is not recommended. More studies need to be carried out to understand the frequency and prevalence of anemia to establish its unique characteristics associated with TB itself. Data regarding the incidence of the disease in high-risk individuals are required for an early detection and a better understanding of anemia in chronic infections such as TB. A multidisciplinary approach including routine follow-ups, regular screening practices, early diagnosis, and medication compliance along with treatment of the underlying cause goes a long way in reducing the incidence of TB-associated anemia.
